# Breaking Down the Barrier: The Role of Cervical Infection and Inflammation in Preterm Birth

**DOI:** 10.3389/fgwh.2021.777643

**Published:** 2022-01-18

**Authors:** Ourlad Alzeus G. Tantengco, Ramkumar Menon

**Affiliations:** ^1^Division of Basic and Translational Research, Department of Obstetrics and Gynecology, The University of Texas Medical Branch at Galveston, Galveston, TX, United States; ^2^Department of Biochemistry and Molecular Biology, College of Medicine, University of the Philippines Manila, Manila, Philippines

**Keywords:** biomarker, cervix, cervical remodeling, pregnancy, parturition

## Abstract

Approximately 40% of cases of spontaneous preterm birth (sPTB) are associated with ascending intrauterine infections. The cervix serves as a physical and immunological gatekeeper, preventing the ascent of microorganisms from the vagina to the amniotic cavity. The cervix undergoes remodeling during pregnancy. It remains firm and closed from the start until the late third trimester of pregnancy and then dilates and effaces to accommodate the passage of the fetus during delivery. Remodeling proceeds appropriately and timely to maintain the pregnancy until term delivery. However, risk factors, such as acute and chronic infection and local inflammation in the cervix, may compromise cervical integrity and result in premature remodeling, predisposing to sPTB. Previous clinical studies have established bacterial (i.e., chlamydia, gonorrhea, mycoplasma, etc.) and viral infections (i.e., herpesviruses and human papillomaviruses) as risk factors of PTB. However, the exact mechanism leading to PTB is still unknown. This review focuses on: (1) the epidemiology of cervical infections in pregnant patients; (2) cellular mechanisms that may explain the association of cervical infections to premature cervical ripening and PTB; (3) endogenous defense mechanisms of the cervix that protect the uterine cavity from infection and inflammation; and (4) potential inflammatory biomarkers associated with cervical infection that can serve as prognostic markers for premature cervical ripening and PTB. This review will provide mechanistic insights on cervical functions to assist in managing cervical infections during pregnancy.

## Introduction

Preterm birth (PTB) is defined as live birth before the completion of 37 weeks of pregnancy (1). It remains a global public health concern, affecting almost 15 million babies annually (2). It is also the leading cause of death in children younger than 5 years of age. Preterm neonates have a greater risk of developing neonatal morbidities, such as respiratory distress syndrome, bronchopulmonary dysplasia, necrotizing enterocolitis, sepsis, and infections (3). Studies have shown that prematurity may also cause a greater risk of developing comorbidities in adulthood, such as cardiometabolic, respiratory, and neuropsychiatric disorders (3–5).

PTB is now recognized as a syndrome associated with multiple pathologic mechanisms, including infection, vascular disorders, uterine overdistension, breakdown in maternal-fetal tolerance, and cervical disease (6). PTB is also associated with socioeconomic, lifestyle, and environmental factors (7–9). Previous studies have shown the association of advanced maternal age, smoking, drinking alcohol, illegal drug use, domestic violence, physical abuse, and exposure to environmental pollutants (7–10). PTB can be medically indicated in women who undergo preterm delivery due to pathologic conditions, such as preeclampsia or intrauterine growth restriction, or spontaneous when the cause is unclear (11, 12). PTB can also be due to preterm premature rupture of membranes (PPROM). However, most PTBs are due to spontaneous PTB with intact membranes (13, 14). It is estimated that 25–40% of PTBs are caused by intrauterine infections (14, 15). Most intrauterine infections during pregnancy are caused by bacteria ascending from the vagina and the cervix (16, 17). How the cervix is compromised to cause the ascent of an infectious agent is still not fully understood.

The cervix plays an essential role in protecting the developing fetus in the intra-amniotic cavity and helps to maintain the pregnancy until term delivery. The cervix remains firm and closed throughout pregnancy and undergoes cervical ripening and dilation during labor and delivery (18, 19). This process is called cervical remodeling and is divided into four distinct but overlapping phases: softening, ripening, dilation, and postpartum cervical repair (20). Cervical remodeling is associated with increased vascular permeability, production of inflammatory cytokines and collagen-degrading enzymes, leukocyte infiltration, and protease activation, which will further degrade the collagen in the cervix (21–26). This will weaken the cervix resulting in the disappearance of its morphological features to allow effacement of the uterus, which will facilitate labor and childbirth.

The cervix also serves as a barrier preventing the ascent of microorganisms from the vaginal canal to the uterine cavity. The mucus produced by cervical epithelia serves as a barrier to prevent infection (27–29). It also protects the cells against chemical and mechanical insults during pregnancy and parturition (30). Cytokines and chemokines secreted by cervical cells recruit and stimulate inflammatory cells and antimicrobial factors to kill invading pathogens (21, 31, 32). These functions of the cervix are partly regulated by the junction proteins that seal off the intercellular space and ensure apical and basolateral polarity. In mouse studies, tight junction proteins, such as claudin 1 and 2 and desmoglein, exhibit temporal changes during pregnancy. These proteins are upregulated during cervical ripening to ensure barrier formation in the cervix, which will prevent infection and other insults in the cervix and the reproductive tract (18, 33).

A compromise in the integrity of the cervical epithelial cell barrier makes the underlying cervical cells more susceptible to infection, which can promote inflammation and predispose women to preterm labor (PTL) (34). Decrease or degradation of these tight junctions might contribute to the pathogenesis of infection mediated PTB (33, 35). The other factors that can alter the integrity of the cervical epithelial barrier and induce premature cervical remodeling include microRNAs, the biomechanical properties of the cervix, and the cervicovaginal metabolome and microbiome (33, 34, 36–38). Conditions that compromise the integrity of the cervix, such as cervical insufficiency, short cervix, cervical trauma, and damage to the cervix from previous surgical treatments for cervical dysplasia, also contribute to PTL and PTB (39–44).

To summarize the evidence on the role of infection and inflammation in the cervix in the pathogenesis of PTB, we conducted this review, which focused on: (1) the epidemiology of cervical infections in pregnant patients; (2) cellular mechanisms that may explain the association of cervical infections to premature cervical ripening and PTB; (3) endogenous defense mechanisms of the cervix to protect the uterine cavity from infection and inflammation; and (4) potential inflammatory biomarkers associated with cervical infection that can serve as prognostic markers for premature cervical ripening and PTB. This review will provide mechanistic insights on cervical functions to assist in the management of cervical infections during pregnancy.

## Cervical Infections Promote Preterm Birth

**Figure 1:**
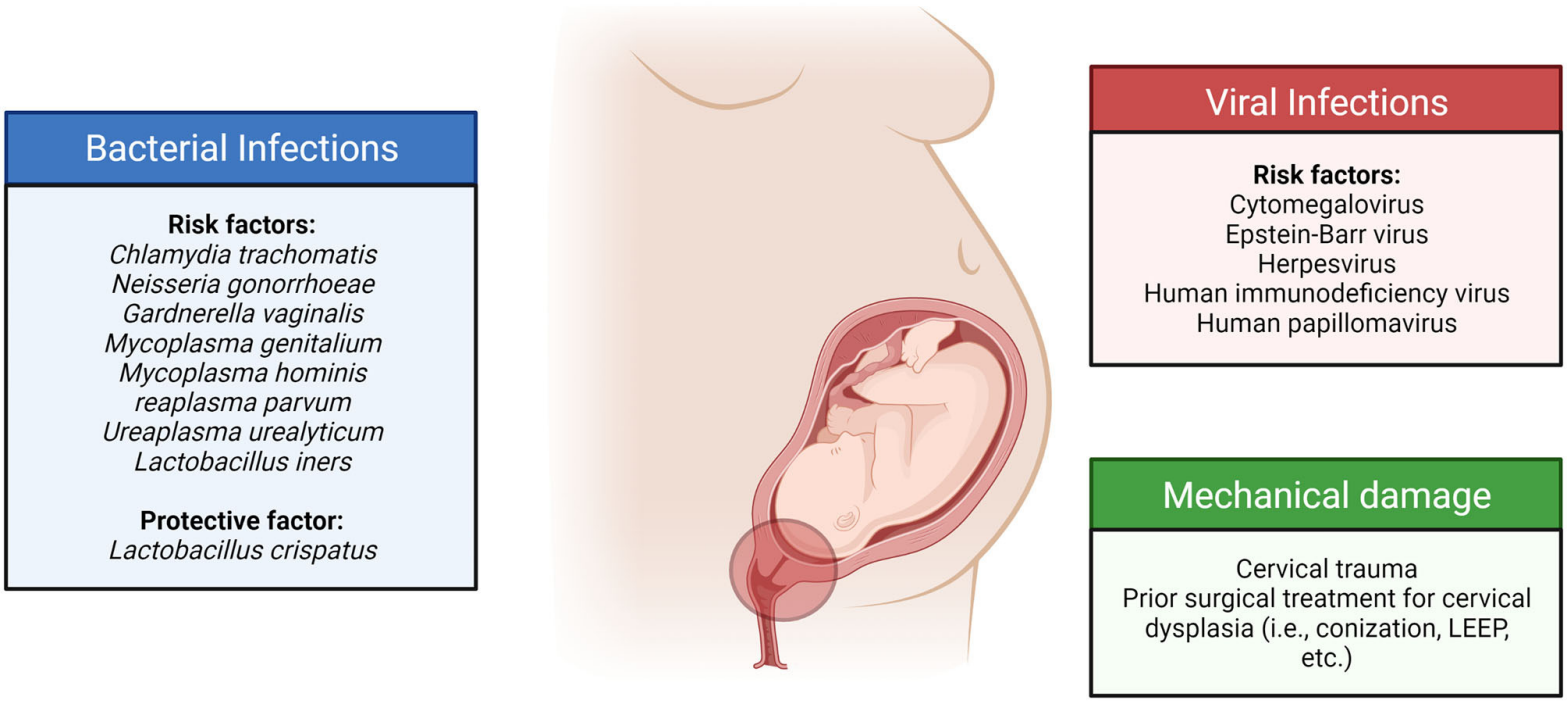
Cervical infections and their sequelae are associated with an increased risk of adverse pregnancy outcomes.

Infection and inflammation in the cervix appear to play a role in pregnancy and parturition. Cervical inflammation, with or without sexually transmitted disease, was significantly associated with PTB (45). We have summarized the current literature on bacterial and viral infections in the cervix during pregnancy and their association with maternal and fetal outcomes (Figure 1).

### Bacterial Infections

Chronic cervical infections with bacteria were associated with high rates of recurrent threatened pregnancy loss, PPROM, and PTB. *Ureaplasma urealyticum* was the most isolated microorganism in the cervix of patients who experienced preterm delivery, with a prevalence of 34.5%, followed by *Enterococcus* spp. (27.6%) and *Mycoplasma hominis* (17.2%). On the other hand, *U. parvum* (85.48%) was the most prevalent pathogen detectable by PCR in the cervix, followed by *Chlamydia trachomatis* (8.0%) (46). Another study also showed that endocervical infection with *Ureaplasma* spp. was significantly associated with PTB (47).

The prevalence of *C. trachomatis* cervicitis in women with PTL was significantly higher in patients with PTBs than in women with term deliveries (48). Another study showed that cervicitis among pregnant women was commonly caused by *C. trachomatis*. Antimicrobial treatment reduced the incidence of preterm delivery in these patients, confirming an infectious etiology (49). In a case-control study in Iran involving 75 women with PTBs and 75 women with term deliveries, no significant association was found between *C. trachomatis* infection of the cervix and PTB (50). Patients with cervical *C. trachomatis* infection treated with antibiotics had lower rates of pPROM, PTB, and small-for-gestational-age infants compared to those who did not receive treatment (51, 52).

Among pregnant patients with PPROM, the presence of the abundant cervical microorganism *Gardnerella vaginalis* was related to microbial invasion of the amniotic cavity (MIAC) (with: 65% vs. without: 44%) (53). A study by Bartkeviciene et al. showed that *Ureaplasma* infections were associated with PPROM, placental inflammation, and newborn respiratory distress syndrome (54). A case-control study involving 94 pregnant Korean women used machine learning tools to predict PTB based on the bacterial risk score of cervicovaginal fluid (CVF). The PTB risk was low when the *Lactobacillus iners* ratio was high. A moderate and high risk was classified when the *L. iners* ratio was low, and the *U. parvum* ratio was high (55).

There were also observed temporal differences in the risk of PTB from cervicovaginal infections. A previous study showed that cervicovaginal infection in the second trimester, when the cervix is neither open nor short, did not significantly raise the rate of PTB. On the other hand, a third-trimester infection significantly increased the risk of PTB (RR: 2.1, *p* = 0.05). When this infection was coupled with premature cervical ripening, the risk of PTB was even more increased (RR: 14.0, *p* = 0.001) (56).

Genital infections during pregnancy are usually polymicrobial. Aside from infections with a single bacterial species, these microorganisms' type, composition, and diversity can affect the cervix and pregnancy outcomes. Abnormal vaginal flora, including *M. hominis*, was associated with cervical shortening. This compromises the cervix and exposes the intrauterine cavity to the pathogens in the cervicovaginal area, increasing PTB risk (57). In a previous microbiome study involving pregnant women of predominantly Caucasian ethnicity, CST III (*L. iners*-dominated) was also associated with extreme cervical shortening (OR: 6.4, 95% CI: 1.32–31.03) (58). Pregnant women with a cervicovaginal community characterized by increased facultative anaerobes and decreased *Lactobacillus* species (CST IV) are associated with short cervix and PTB (59). However, the presence of *L. crispatus*-dominated cervical microbiota in women with PPROM was associated with a lower risk of intra-amniotic complications and early-onset sepsis of the newborn (60). This lowered risk of complication from PPROM can be attributed to the antimicrobial activity of *L. crispatus* and other probiotics against bacterial vaginosis and urinary tract infection pathogens (61, 62).

In summary, these data showed the association of cervical infection with increased risk PTB. Moreover, antibiotic treatment of cervical infections resulted in better pregnancy outcomes. Some studies showed an association between single-species cervical infection, such as *C. trachomatis, Enterococcus* spp., *G. vaginalis, M. hominis, U. parvum*, and *U. realyticum*, and PTB. However, other studies also showed the importance of polymicrobial infection, bacterial composition, and diversity in increasing the risk of PTB. Cervical microbiota dominated by facultative anaerobes and with decreased *Lactobacillus* species were more predisposed to PTB.

### Viral Infections

Viral infections in the cervix are also documented to impact pregnancy outcomes. Systemic infections, such as HIV, were also associated with cervical inflammation and prematurity. In a previous study involving pregnant women living with HIV-1 infection (PWLWH), 12% of them delivered preterm. PWLWH had a higher prevalence of *L. iners* dominant and mixed anaerobes groups, which were associated with increased pro-inflammatory cytokine concentrations (such as IL-1b) in the CVF (63). Epstein-Barr virus (17.2%) was the most prevalent pathogen detectable by PCR in the cervix of patients with PTB, followed by human herpesvirus 6 (8.0%) (46). In a study involving Kenyan women, cervical cytomegalovirus (CMV) infection was associated with higher cervical IL-6 and TNF-a. However, this did not significantly increase PTBs before 34 weeks of gestation (64). Genital herpes infection can cause blistering and ulceration of the cervix. This can disrupt the cervical epithelial barrier and promote ascending bacterial infections during pregnancy (65). A study involving 662,913 mother-newborn pairs showed that genital herpes during the first or second trimester was associated with more than double the risk of PTB (66).

Human papillomavirus (HPV) infection was also prevalent among pregnant patients. The estimated HPV prevalence in pregnant women varied from 9.58 to 46.67%, with a summary estimate of 16.82% (95% CI: 16.21–17.47) (67). The prevalence varied based on study region, age, and HPV type. Previous reports have shown that HPV infections are associated with an increased risk of adverse pregnancy outcomes (68–71). HPV infection is associated with a 1.5-times increased odds of PTB, 1.96-times increased odds of PPROM, 1.91-times increased odds of low birth weight, and 2.23-times increased odds of fetal death (70). A retrospective study involving 2,153 pregnant women showed an HPV positivity rate of 38.5%. HPV infection (OR: 2.07, 95% CI: 1.03–4.14) was associated with PPROM. HPV infection was also associated with newborn septicemia, respiratory distress syndrome, neonatal intensive care unit admission, and low birth weight (68). A retrospective population-based study showed that HPV infections before or during pregnancy were associated with PTB [adjusted odds ratio (aOR): 1.19, 95% CI: 1.01–1.42], PPROM (aOR: 1.52, 95% CI: 1.18–1.96), premature rupture of membranes (aOR: 1.24, 95% CI: 1.08–1.4), and neonatal mortality (aOR: 2.69, 95% CI: 1.25–5.78) (71).

Lack of intervention during chronic HPV infections can result in cervical intraepithelial neoplasia (CIN) and cervical cancer. Based on a previous study from the Scottish HPV archive, HPV-associated high-grade cervical diseases (CIN2 and CIN3) but not high-risk HPV infection or low-grade cervical disease (CIN1) were associated with PTB (OR: 1.843, 95% CI: 1.101–3.083) (72). Cervical surgeries that are usually done to treat chronic infections in the cervix were shown to be associated with adverse pregnancy outcomes. Maternal history of cervical conization and/or loop electrical excision procedure (LEEP) increases the risk of preterm delivery, irrespective of concurrent maternal HPV positivity (73). Another study showed that previous treatment for CIN from chronic HPV infection was associated with even more significant risks for PTB and PPROM and was also associated with PROM, neonatal mortality, and maternal and neonatal infectious complications (71).

A meta-analysis showed an overall higher risk of PTB associated with excisional procedures (RR: 1.87, 95% CI: 1.64–2.12) than ablative procedures (RR: 1.35, 95% CI: 1.20–1.52) in the cervix. The risk of PTB was higher for more radical excisional techniques, such as cold knife conization (RR 2.70, 95% CI 2.14–3.40), laser conization (RR: 2.11, 95% CI: 1.26–3.54), and large loop excision of the transformation zone (RR: 1.58, 95% CI: 1.37–1.81) (74). In summary, these results show that viral infections in the cervix, complications resulting from such conditions, and associated surgical procedures to treat them increased PTB risk.

## Infection, Inflammation, and Cervical Remodeling

**Figure 2:**
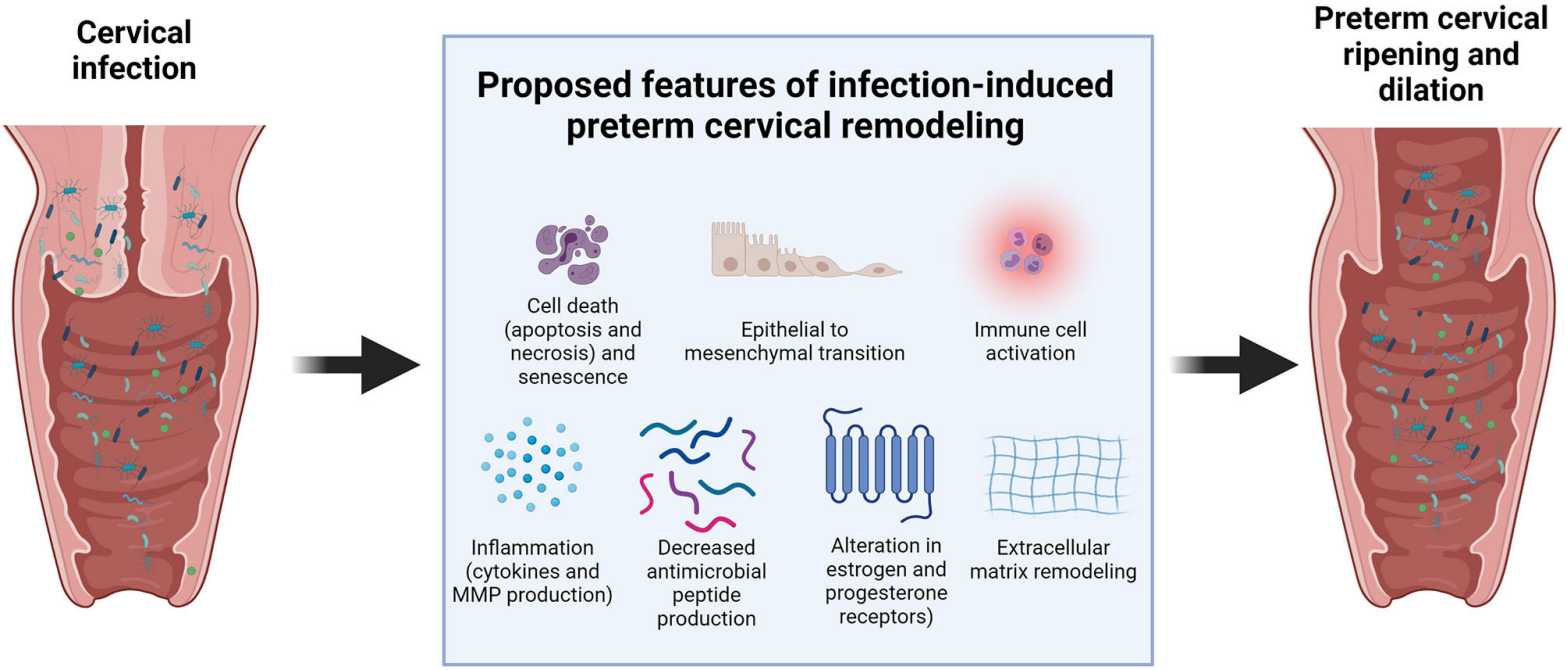
Proposed cellular mechanisms involved in infection-induced preterm cervical remodeling.

Cervical infection and inflammation may damage the cervix and hasten the cervical remodeling process during pregnancy. This aberrant timing of cervical ripening can increase the risk of PTB. Previous clinical, animal, and *in vitro* studies suggested several potential mechanisms on how infections can compromise the integrity of the cervix (Figure 2). Cervical canal infection with *Ureaplasma, Mycoplasma*, and *Escherichia coli* in pregnant women with singleton pregnancies was associated with cervical changes, which could be easily detected by the Bishop score (a pre-labor scoring system based on a digital cervical exam of the patient, which utilizes cervical dilation, position, effacement, consistency of the cervix, and fetal station to assist in predicting whether induction of labor will be required) and ultrasound assessment of cervical length (75, 76). However, another study showed no significant association between vaginitis, cervicitis, and cervical length (77).

Limited animal studies showed the potential mechanisms of how bacterial and viral infections can promote cervical ripening in the cervix. Sierra et al. reported that colonization of the cervicovaginal space with *Gardnerella vaginalis* significantly increased IL-6 levels in the cervicovaginal space. It also increased the gene expression of IL-1β, IL-8, and IL-10 in the cervix. Soluble E-cadherin, a molecular marker of cervical epithelial barrier disruption, and Tff-1, a molecule involved in cervical remodeling, were also increased with *G. vaginalis* infection. It also altered the cervical biomechanics, as shown by the significant decrease in tissue modulus of cervices colonized by *G. vaginalis* compared to uninfected cervices (78). This indicates that colonization hastens the cervical softening process.

McGee et al. reported that cervical HSV-2 infection promoted cervical remodeling in a mouse model of pregnancy. HSV-2 induced collagen remodeling and increased hyaluronic acid synthesis. HSV-2 also altered the responsiveness of the cervix to pregnancy hormones by changing the expression of estrogen and progesterone receptors in the cervical epithelium, which promoted premature cervical ripening and increased the risk of PTB (79). Cervical viral infections can also make the cervix more susceptible to bacterial infections that are more pathogenic and can cause PTB. Murine gammaherpesvirus 68 infections resulted in a lower expression of antimicrobial peptides (AMPs) and lower levels of pro-inflammatory cytokines in the cervix compared to pregnant mice not exposed to the virus (80).

Our previous *in vitro* studies have shown that infection and inflammation can cause cell-specific changes that contribute to cervical damage and cervical remodeling during pregnancy. These include inflammation, epithelial to mesenchymal transition (EMT), cell death (apoptosis, necrosis, and autophagy), and senescence. LPS and TNF-a increased the levels of inflammatory cytokines (IL-6 and IL-8) and MMP-9 in ectocervical and endocervical epithelial cells in *in vitro* monoculture and co-cultured in a microfluidic organ-on-a-chip of the cervical epithelial layer (34, 81). This increase in collagenolytic enzymes and pro-inflammatory cytokines can disrupt the cervical epithelial barrier and promote cervical ripening (20, 82, 83). Apoptosis and necrosis in the resident cervical cell types may contribute to cervical tissue remodeling during pregnancy. Human cervical stromal biopsies from pregnant women in active labor have shown higher apoptotic nuclei than those from cervices not in labor (84). Infection and inflammation also increased apoptosis and senescence in cervical epithelial cells (81). Similarly, sterile inflammation from oxidative stress also promoted an increased inflammatory response, apoptosis, senescence, and autophagy in cervical epithelial and stromal cells (85).

We have also shown that infection and inflammation can promote EMT in cervical epithelial cells (81, 86, 87). EMT was shown to be involved in the cervical changes that happen during parturition. Human and animal studies showed an increase in the expression of mesenchymal markers, such as vimentin and N-cadherin, and a decrease in epithelial markers, such as cytokeratin and E-cadherin, in the cervix during parturition (88, 89). EMT is often associated with an inflammatory milieu, primarily to remodel the affected area; however, a non-reversible state of EMT induced in response to infection or other risk exposures may cause persistent inflammation and cervical matrix degradation.

Aside from infection and inflammation, chemical and mechanical damage to the cervix can also promote inflammation resulting in premature cervical ripening. A previous animal study showed that pre-gestational cervical excision plus inflammation-induced PTL significantly increased the expression levels of collagen type IV alpha 1, collagen type V alpha 1, and MMP-14. This increase may promote premature cervical remodeling and PTB (90). A clinical study also showed that vaginal or cervical infections only demonstrated an increased risk for PTB in patients with a short cervix before 28 weeks or dilated cervix before 37 weeks of pregnancy (56). A recent study showed that damage to the cervical epithelium induced by a common spermicide, nonoxynol-9, promoted ascending infections with *U. parvum*, upregulated the production of pro-inflammatory cytokines, and increased PTB rates in mice (30). Altogether, these data show that an intact cervix can effectively prevent ascending infections in pregnancy. Unless the cervix is compromised by previous surgical procedures, chemical exposure, or overt infection, it can continue its role in protecting the fetus until term delivery.

## Endogenous Defense Mechanisms of the Cervix Against Infections During Pregnancy

The cervix has endogenous defense mechanisms to fight off infections during pregnancy. These serve as the first line of defense and help maintain the cervix's barrier function during pregnancy. The cervical epithelial layers form a barrier through its junction proteins that seal off intercellular spaces and block the bacterial invasion of the cervix (18, 33). The cervix also produces AMPs that can kill bacteria. In a previous study in the United Kingdom, cervical fluid elafin or human β-defensin-1 protein levels were not associated with PTB (91). Another study showed that low β-defensin-2 protein levels (aOR: 1.40, 95% CI: 0.83–2.34) had slightly elevated but not significantly increased odds of sPTB (92). Another study showed that cervical gene delivery of HBD3 may be a potential candidate to boost innate immunity in the cervix and prevent *E. coli* ascending infection related PTB and its associated neonatal consequences (93).

As mentioned above, the cervical mucus plug (CMP) also plays a vital role in deterring bacteria from the vaginal canal trying to get past the cervix to invade the uterine cavity. The CMP contains large amounts of AMPs, such as secretory leukoprotease inhibitor (SLPI), lysozyme, lactoferrin, calprotectin, α-defensin human neutrophil peptides (HNPs) 1–3 and human β-defensin (27). Previous studies have shown that cervical mucus has good antibacterial properties. It can completely inhibit pathogenic bacteria such as *Staphylococcus saprophyticus, E. coli, Pseudomonas aeruginosa, Enterococcus faecium, Streptococcus agalactiae, S. pyogenes*, and *S. aureus* (94–96).

The CMP also performs essential innate and adaptive immune functions. CMP proteins can also activate the innate immune response of the cervix to kill bacteria trying to invade the cervix (97). Hence, a compromise in the barrier properties of the cervical mucus can predispose pregnant patients to PTB. Critchfield et al. reported that women at high risk for PTB have mucus that is more extensible and permeable and forms weaker gels than cervical mucus from women at low risk for PTB (28).

The cervix increases pro-inflammatory cytokine production to help fight off infection. Pregnant women with bacterial vaginosis or cervicitis have higher levels of IL-1β, IL-6, and IL-8 in their cervical mucus compared to patients with normal pregnancies (98–100). Bacterial and viral infections can also elicit a type 1 T-helper lymphocyte response in the cervix. This regulates cytotoxic T lymphocytes, which are in part responsible for clearing infections in the genital tract (101). Massive inflammation in the cervix may be detrimental since this can damage the cervical epithelial barrier (34, 37). However, the cervix also modulates the inflammatory response to an infection via secreting anti-inflammatory cytokines. Dubicke et al. showed that the anti-inflammatory cytokine IL-4 was significantly higher in the preterm subgroup with bacterial infection than in the non-infected group (21).

The cervix also harbors immune cells that can destroy pathogenic bacteria and viruses. Some of the primary immune cells in the cervix include macrophages, dendritic cells (DCs), and other lymphocyte lineage-negative cells (CD3, CD20, CD56, and CD16) (102). These immune cells are activated by bacteria and viruses that infect the cervix. Prakash et al. reported that women with cervicitis had increased recruitment of macrophages in their cervical epithelium (103). Endocervical secretions of patients with chlamydial cervicitis had higher CD4+, CD8+, and DC phenotypes (104). This shows that the cervix mounts a cell-mediated immune response to *C. trachomatis*. Persistent HSV-2 infection results in the formation of lymphoid clusters of CD4+ and CD8+ T-cells, B-cells, DCs, and macrophages in the cervix (105). These lymphoid cells persist for years, even after viral clearance, and confer lasting protection against reinfection (106). In summary, these physical, chemical, and immunological defense mechanisms in the cervix may help prevent the pathologic effects of bacterial and viral infections in the cervix. These mechanisms enable the cervix to perform its barrier function to protect the developing fetus in the uterine cavity.

## Potential Cervical Biomarkers of Adverse Pregnancy Outcomes

The type of cervical infection and the specific cellular and immunologic responses of the cervix against infections can be used as biomarkers for diagnosing infections and prognosticating pregnancy outcomes (98, 100). Previous studies have shown some potential biomarkers associated with cervical infections that may be useful in predicting adverse pregnancy outcomes. In pregnancies with PTL, both intra-amniotic infection and sterile inflammation were associated with elevated concentrations of IL-6 in the cervical fluid (intra-amniotic infection: median 587 pg/mL; sterile intra-amniotic inflammation, median: 590 pg/mL; without inflammation, median: 136 pg/mL) (107). IL-6 and IL-8 in cervical secretions can also predict PTL caused by intra-amniotic infection or chorioamnionitis (108). Kim et al. showed that IL-8 and MCP-1 in the CVF were significantly increased in patients with intra-amniotic infections and/or inflammation (55).

Macrophage inflammatory protein (MIP)-1β levels in CVF were significantly higher in patients with imminent sPTB than those with term deliveries (55, 109). However, another study showed that the concentration of MIP-1α in the cervical fluid was not elevated in patients with PTL who had intra-amniotic infections. However, elevated MIP-1α levels in amniotic fluid may be valuable markers for intra-amniotic infection in women with PTL (107). The utility of amniotic fluid markers is limited, as many perinatal clinical settings around the globe do not perform amniocentesis for diagnostic purposes.

In women with PTL, elevated CVF levels of C5a (a complement-activated product) and IGFBP-1 (a transport protein for insulin-like growth factor 1) were significantly associated with IAI and PTB at <34 weeks of gestation, while those of C3a were associated with IAI but not PTB. Compared to amniotic fluid white blood cells, these biomarkers have similar or better diagnostic performance (110). In a study involving 35 women with PTB in the United Kingdom, IL-8 and IL-1β in the cervical fluid were lower in women who delivered preterm than those who had term delivery (91).

In a retrospective study of 99 women with PPROM, the use of protein-antibody microarray technology identified biomarkers in the CVF that can identify MIAC in women with PPROM. These biomarkers include IL-8, lipocalin-2, MIP-1α, MMP-9, and TIMP-1 (111). The protein concentrations of IgGFc-binding protein (FcγBP), which is a core mucus protein with anti-inflammatory properties, in the cervical fluid were also elevated in the presence of intra-amniotic infection in patients with PPROM (presence: 345 ng/mL vs. absence: 60 ng/mL) (112). This increase in FcγBP may help prevent massive inflammation from intra-amniotic infections.

AMPs and metabolites in the CVF may also serve as biomarkers for PTL and PTB. The cervicovaginal level of trappin-2, an AMP and a serine protease inhibitor, was found to be elevated in women who delivered preterm (113). A metabolomic study showed that high acetate levels in the CVF of women with PTL were predictive of preterm delivery and delivery within 2 weeks of presentation with PTL (109). Moreover, the predictive accuracy of acetate levels in CVF for PTB in symptomatic pregnant women was comparable to cervical length and fetal fibronectin (114). These data showed the potential use of cervical samples in developing biomarkers for stratifying pregnant patients at greater risk of PTB.

## Clinical Implications

Cervical infections remain underdiagnosed among the pregnant population because patients are generally asymptomatic. This results in a chronic subclinical infection extending to the upper genital tract and a greater risk of adverse pregnancy outcomes. Currently, screening tests for cervical infections are not routinely done in most hospitals. The gold standard for diagnosing cervical infections is the nucleic acid amplification test. However, this is not available in most hospitals, especially in developing countries. This results in many pregnant women not being diagnosed with cervical infections. A previous study involving 1,876 pregnant women in Japan noted that 84% of pregnant women with gonococcal cervicitis may have been missed in hospitals that do not routinely perform the screening test for gonococcal cervicitis (115). These pregnant women who are not diagnosed with cervical infection are also not given the proper management that can help in preventing ascending infections during pregnancy.

Aside from the challenges in diagnosis, there are still a lot of uncertainties in managing cervical infections during pregnancy. The CDC has recommendations for managing common cervical infections during pregnancy, such as chlamydia, gonococcal, and *M. genitalium* infection (116). However, more studies are needed to manage non-chlamydial and non-gonococcal cervicitis, because these were shown to have an association with adverse pregnancy outcomes. The limited available data showed that antibiotics directed toward *C. trachomatis* and *Neisseria gonorrhoeae* are ineffective against non-chlamydial and non-gonococcal cervicitis (117).

Several cervical infections can easily be prevented by patient education and vaccination. The acquisition of many STIs is based on individual sexual behavior. Educating patients on safe sex practices can help prevent cervical infections (118). Vaccines are already available for HPV infection. Increasing the knowledge and awareness of patients regarding the HPV vaccination and improving its coverage in women may help prevent HPV infections during pregnancy.

## Conclusion

This review summarized the existing evidence on the association of cervical infection and inflammation and PTB. Multitudes of studies have reported several biomarkers in response to infection that are pro-inflammatory that can cause derangement in the cervical environment. However, the cervix may withstand exogenous insults due to multiple barriers (mechanical barriers: tight junctions that seal the cervical epithelial layer and the CMP that blocks invading pathogens from passing through to the uterine cavity; chemical barriers: production of AMPs and anti-inflammatory cytokines that can fight off infection and temporize inflammation; and immunologic barriers: innate and adaptive immune responses in the cervix) that protect the cervix and balance the impact of infection and inflammation.

However, overt infections with a high load of pathogenic bacteria and viruses may overcome these barriers, compromise the barrier function of the cervix, and eventually increase the risk of PTB. Infection disrupts the integrity of the cervical epithelial barriers by promoting cell death and senescence, EMT, and decreasing AMP production. When the cervical epithelial cell layer is damaged, microorganisms can access and infect the stromal layer and cause collagen remodeling and degradation. Infections have been shown to promote collagen degradation by increasing the levels of collagenolytic enzymes and decreasing tissue inhibitors of matrix metalloproteinases. Cervical infection can also promote an inflammatory response, immune cell activation, and alteration of estrogen and progesterone receptors. These events will weaken the cervix and allow microorganisms to invade the uterine cavity, infect the developing fetus, and cause PTB.

## Author Contributions

OT and RM conceived the review, contributed to analysis and interpretation of available literature, and prepared the manuscript. Both authors contributed to the article and approved the submitted version.

## Funding

OT is an MD-PhD trainee in the MD-PhD in Molecular Medicine Program, supported by the Philippine Council for Health Research and Development, Department of Science and Technology, Republic of the Philippines, and administered through the University of the Philippines Manila. This study was supported by 5R01HD100729-03 (NIH/NICHD) to RM.

## Conflict of Interest

The authors declare that the research was conducted in the absence of any commercial or financial relationships that could be construed as a potential conflict of interest.

## Publisher's Note

All claims expressed in this article are solely those of the authors and do not necessarily represent those of their affiliated organizations, or those of the publisher, the editors and the reviewers. Any product that may be evaluated in this article, or claim that may be made by its manufacturer, is not guaranteed or endorsed by the publisher.
